# Effects of syphilis infection among HIV-1-positive individuals on suppressive antiretroviral therapy

**DOI:** 10.1186/s12981-022-00493-w

**Published:** 2022-12-31

**Authors:** Phillip Chan, Tommy H. C. Tang, Ruby T. S. Kwong, Lawrence Chan, Helen S. Y. Chan, K. W. Lam, W. M. Ting, S. K. Yung, Emily C. T. Lam, M. Y. Chu, Wilson Lam, T. C. Wu, Patrick Li, M. P. Lee

**Affiliations:** 1 Department of Medicine, Queen Elizabeth Hospital, Hong Kong, China; 2 Institute of HIV Research and Innovation, Bangkok, Thailand; 3 Hong Kong Sanatorium & Hospital, Hong Kong, China

**Keywords:** HIV/syphilis co-infection, CD4 decline, Lymphocyte depletion, Sexually transmitted infection, Viral suppression

## Abstract

**Introduction:**

Incident syphilis leads to changes in plasma HIV-1 RNA and CD4 + T-cell level in people with HIV (PWH) with viraemia. Its effect in PWH on suppressive antiretroviral therapy (ART) is less clear.

**Methods:**

PWH on suppressive ART (plasma HIV-1 RNA < 50copies/mL) followed at the Queen Elizabeth Hospital, Hong Kong, China were regularly screened for syphilis. Their plasma HIV-1 RNA, CD4 + and CD8 + T-cell, and total lymphocyte levels before syphilis, during syphilis, and after successful treatment were compared.

**Results:**

Between 2005 and 2020, 288 syphilis episodes from 180 individuals were identified; 287 episodes were related to male, with a median age of 41 at diagnosis; 221 (77%) were syphilis re-infection. The rates of plasma HIV-1 suppression were statistically unchanged across the time-points (97% pre-syphilis, 98% during syphilis, and 99% post-treatment).

Total lymphocyte, CD4+ and CD8+ T-cell levels decreased during incident syphilis (p<0.01), and rebounded post-treatment (p<0.01). VDRL titre was associated with declines in CD4+ T-cell (p=0.045), CD8+ T-cell (p=0.004), and total lymphocyte levels (p=0.021). Pre-syphilis CD4/CD8 ratio was associated with increases in CD8+ T-cell (p=0.001) and total lymphocyte levels (p=0.046) during syphilis. Syphilis re-infection was associated with an increase in total lymphocyte level (p=0.037). In the multivariable analysis, only pre-syphilis CD4/CD8 ratio was independently associated with increases in CD8+ T-cell (p=0.014) and total lymphocyte levels (p=0.039) during syphilis.

**Conclusions:**

Among virally-suppressed PWH, total lymphocyte, CD4+, and CD8+ T-cell levels declined during incident syphilis but rebounded post-treatment. The status of plasma HIV suppression was unaffected by syphilis.

**Supplementary Information:**

The online version contains supplementary material available at 10.1186/s12981-022-00493-w.

.

## Introduction

*Treponema pallidum* infection (syphilis) is a re-emerging sexually transmitted infection (STI) worldwide and is common among people with HIV-1 (PWH) and men who have sex with men (MSM) [1, 2]. Syphilis is known to worsen HIV-1 infection. During incident syphilis, plasma HIV-1 RNA increases and CD4 + T-cell level declines in untreated PWH [3–5]. PWH also experience a higher rate of syphilis treatment failure than HIV-negative populations [6, 7]. The effects of syphilis on HIV-1 in PWH on suppressive antiretroviral therapy (ART) are less clear. In a study of 84 PWH, of which 80% were on suppressive ART, early syphilis was associated with a significant reduction in the total lymphocyte level and levels of lymphocyte subsets including CD4 + T cells [8]. While plasma HIV-1 RNA level was unaffected by syphilis in that study [8], higher risk of plasma HIV-1 RNA elevation and decline of CD4 + T-cell level were observed in another study with participants receiving ART [5].

Given the global resurgence of syphilis and the emergence of azithromycin-resistant *Treponema pallidum* subspecies, investigation into the effect of syphilis on HIV-1 control is of public health importance. This study aimed to examine the effects of syphilis in PWH on suppressive ART. The levels of CD4 + and CD8 + T-cells and total lymphocyte count before, during and after syphilis from a group of PWH with pre-existing plasma viral suppression were compared. The final aim was to identify HIV- and syphilis-related determinants of CD4 + and CD8 + T-cell count and total lymphocyte level.

## Methods

### Study design and participants

This retrospective, single-centre cohort study was based on the clinical and laboratory records of 3,249 PWH who attended out-patient HIV service at Queen Elizabeth Hospital (QEH) in Hong Kong between 2005 and 2020. They were followed up at the clinic every 3 to 4 months and were regularly screened for syphilis in parallel with routine laboratory testing. The laboratory tests included complete blood count, plasma HIV-1 RNA level (Siemens VERSANT® HIV-1 RNA 3.0 Assay before 2011 and Roche, COBAS AmpliPrep HIV-1 after 2011), blood CD4 + and CD8 + T-cell counts determined by flow cytometry. Extra screenings were ordered upon presentation of symptoms suggestive of new syphilis. Syphilis screening followed the reverse sequence approach in which venereal disease research laboratory (VDRL) titre was measured after a positive syphilis enzyme immunoassay (EIA) (Abbott, Architect Syphilis TP) test. Syphilis was defined by a 4-fold rise in VDRL titre or a new seroconversion of EIA test with recent high-risk exposures documented by the attending clinicians.

To examine the effects of syphilis on HIV-1 during suppression ART, laboratory records during incident syphilis from individuals with pre-existing plasma viral suppression (i.e. at least one record of plasma HIV-1 RNA < 50 copies/mL before incident syphilis) were selected. The laboratory outcomes prior to the syphilis diagnosis (T1), at the time of diagnosis (T2), and after successful treatment (T3) were compared. Individuals who had underlying opportunistic infection on treatment or did not have paired plasma HIV RNA, CD4 + and CD8 + T-cell counts throughout T1 to T3 were excluded. Successful syphilis treatment was defined by a 4-fold decline in VDRL titre by 12 months for early syphilis and 24 months for late syphilis. The study protocol was reviewed and approved by the Kowloon Central and Kowloon East Cluster Research and Ethics Committee, Hospital Authority (Ref.: KC/KE-19-0057/ER-4).

### Statistical analysis

Results were reported as median and interquartile range (IQR) or number and percentage, as appropriate. Sequential parameters in continuous and categorical format were compared by Wilcoxon and McNemar test, respectively. Changes in CD4+, CD8 + T-cell, and total lymphocyte counts after syphilis were determined by their differences between T2 and T1 (i.e. T2–T1). The association between the aforementioned changes and various clinical parameters was examined by non-parametric Spearman correlation and Mann-Whitney U test. Factors with a p-value ≤ 0.1 in the univariable analysis were included in multivariable analysis. A two-tailed p-value < 0.05 were considered significant. Statistical analyses were performed using SPSS Version 23.0 (IBM Corp., Armonk, NY).

## Results

Between 2005 and 2020, 288 syphilis episodes from 180 individuals on suppressive ART were identified; 103 (57%) of them had one episode of syphilis, 55 (31%) had two, 15 (8%) had three, 5 (3%) had four and 2 (1%) had five. All but one of the episodes occurred in males. The median age by syphilis episodes was 41 (IQR 33–48) years. Among all 288 episodes, 221 (77%) of them were syphilitic reinfection denoted by a positive Treponemal-specific test at T1 prior to incident syphilis. The median duration between T1 and T2 was 168 (IQR 110–210) days and the duration was less than a year in 279 (97%) of the episodes. The median durations from T2 to treatment and treatment to T3 were 16 (IQR 11–39) days and 116 (IQR 84–175) days respectively. Intramuscular penicillin treatment was given in the majority of syphilis episodes (n = 258, 90%) (Table 1).

### Changes of laboratory parameters across syphilis episode

The median CD4 + T-cell levels (cells/mm^3^) decreased from 559 (IQR 405–699) before syphilis at T1 to 519 (IQR 390–687) at the time of syphilis diagnosis at T2 (p < 0.001) and rebounded to 562 (IQR 430–725) after successful syphilis treatment at T3 (p < 0.001). The median CD8 + T-cell level (cells/mm3) decreased from 822 (IQR 621–1047) at T1 to 733 (IQR 547–954) at T2 (p < 0.001), and rebounded to 805 (IQR 621–1044) at T3 (p < 0.001). The resultant CD4/CD8 ratio increased from 0.67 (IQR 0.50–0.90) at T1 to 0.71 (IQR 0.52–0.95) at T2 (p < 0.001) but remained statistically similar from T2 to T3 (0.70(IQR 0.52–0.95), p = 0.542). The rates of plasma HIV-1 suppression were 97% at T1, 98% at T2, and 99% at T3, revealing statistically similar rates between T1 and T2 (p = 0.791) and between T2 and T3 (p = 0.754) (Table 2).

The median total white blood cell count (WBC) and total lymphocyte count (10^3^ cells/µL), based on the complete blood count, between T1 and T3 were compared. The total WBC levels were 5.9 (IQR 5.1–7.2) at T1, 6.2 (IQR 5.1–7.2) at T2, and 6.1 (IQR 5–7.4) at T3. The levels were statistically similar between T1 and T2 (p = 0.557) and between T2 and T3 (p = 0.970). The median total lymphocyte levels were 2.1 (IQR 1.7–2.6) at T1, 1.9 (IQR 1.6–2.4) at T2, and 2.1 (IQR 1.8–2.6) at T3, revealing a statistically significant decrease from T1 to T2 (p < 0.001) and a significant increase from T2 to T3 after successful syphilis treatment (p < 0.001).

Potential determinants of the changes in CD4+, CD8 + T-cell levels, and total lymphocyte level from T1 to T2, including age, nadir CD4 + T-cell level, CD4/CD8 ratio at T1, VDRL titre, and syphilis reinfection, were compared using non-parametric tests (Additional file [1]). Among them, higher VDRL titre was weakly associated with negative changes in CD4 + T-cell (r(286)=-0.118, p = 0.045), CD8 + T-cell (r(286)=-0.167, p = 0.004), and total lymphocyte levels (r(285)=-0.136, p = 0.021). CD4/CD8 ratio at T1 was weakly associated with positive change of CD8 + T-cell (r(286) = 0.189, p = 0.001) and total lymphocyte (r(285) = 0.118, p = 0.046) levels. Syphilis re-infection (vs. first-ever infection) was associated with lesser decline in total lymphocyte level (-0.1 [IQR − 0.4 to 0.1] vs. -0.3 [IQR − 0.7 to 0], p = 0.037). In the multivariable analysis, CD4/CD8 ratio at T1 was the only independent predictor of a positive change of CD8 + T-cell (unstandardized coefficient (B) = 112.8; 95%CI 22.9–202.7, p = 0.014) and total lymphocyte (B = 0.176; 95%CI 0.009–0.342, p = 0.039) levels while VDRL titre and syphilis reinfection were not.

## Discussion

In this retrospective study with PWH on suppressive ART, CD4 + T-cell level declined after incident syphilis and recovered after successful syphilis treatment. The findings are consistent with existing studies of participants with inconsistent treatment status [4, 5], suggesting that syphilis-related CD4 + T-cell depletion takes place regardless of HIV-1 suppression status. Our study also reveals a similar pattern of change in CD8 + T-cell and total lymphocyte during syphilis and after successful treatment. These two parameters were not thoroughly examined in older studies. In two recent studies of HIV/syphilis co-infection [8, 9], one observed an insignificant decline in CD8 + T-cell during syphilis [9] and both observed a significant increase in CD8 + T-cell level after successful syphilis treatment [8, 9]. One of the studies also demonstrated that B-cells similarly declined after syphilis and rebounded after successful treatment [8]. Together with the similar pattern of change in total lymphocyte in current study, incident syphilis likely leads to non-subset-specific lymphocyte depletion. Further, whether such depletion takes place regardless of HIV-status remains unclear as lymphocyte changes are rarely investigated in HIV-negative populations. In one report, CD4 + T-cell depletion was detected during incident syphilis regardless of HIV status [10].

Higher VDRL titre during syphilis was associated with a greater decline in total lymphocyte, CD4+, and CD8 + T-cell levels in the univariable but not the multivariable analysis. One possibility is that the VDRL titre has to be adjusted with the actual duration between syphilis transmission and diagnosis as VDRL titre usually decays over-time after peaking at primary infection. As it is not feasible and imprecise to figure out the exact duration between syphilis transmission and diagnosis retrospectively, duration between T1 and T2 was used instead in the analysis but it was not associated with any one of the lymphocyte changes. Syphilis reinfection was not associated with the lymphocyte changes in the multivariable model. Intriguingly, a higher CD4/CD8 ratio before syphilis was associated with positive change, and hence less decline in CD8 + T-cell and total lymphocyte levels. A recent report described the potential role of syphilis in inducing apoptosis and pyrotosis in CD4 + and CD8 + T-lymphocytes by altering the intracellular expression of caspase-1 and caspase-3 and their levels in the circulation [11]. Reversal of CD4/CD8 ratio is a measure of immunosenescence and reflects T-cell dysfunction in both HIV-positive and HIV-negative populations [12]. PWH frequently show persistent reversal of CD4/CD8 ratio despite years of HIV-1 suppression [13, 14]. Future studies should examine whether CD4/CD8 ratio modifies cellular response against syphilis and alters the degree of lymphocyte depletion.

In addition to CD4 + T-cell depletion, previous studies also reported a transient increment of plasma HIV-1 RNA during incident syphilis. In this study with participants on stable ART, the frequency of HIV-1 suppression status remained unchanged. The finding suggests that HIV-1 suppression through effective ART is unaffected by syphilis, supporting the notion that lymphocyte depletion during syphilis is unrelated to HIV-1 replication. As plasma HIV-1 RNA level is closely related to the viral shedding in other bodily fluids [15, 16], the stable HIV-1 suppression status also suggests that secondary HIV-1 transmission is unaffected by syphilis in the presence of effective ART. Of note, syphilis reinfection may present with a different immunological profile compared to first-ever infection. In our study, we found a weak association between lesser decline in lymphocyte levels and syphilis reinfection. In another study of 11 individuals, syphilis reinfection was found to present with lower concentrations of plasma interferon-α and chemokine (C-C motif) ligand (CCL) 4 [17].

This study has several limitations. Despite a good sample size, it is a single centre study and the data is based primarily on male participants. The study is retrospective and does not include HIV-negative control. We are unable to determine the stage of syphilis at diagnosis which may alter the laboratory outcomes. Another limitation is the lack of detailed lymphocyte-subsets data in our analysis, we are therefore unable to confirm whether syphilis causes non-subset-specific lymphocyte depletion as reported in the previous study. Lastly, potentially undiagnosed, concomitant STIs may also play a role in the examined parameters.

## Conclusion

In PWH on suppressive ART, incident syphilis leads to a transient decline in CD4 + T-lymphocyte level. Moreover, it appears to affect all subsets of lymphocytes non-specifically. Nonetheless, incident syphilis does not affect the stability of plasma viral suppression. The study observed a high frequency of syphilis reinfection, highlighting the need of regular STIs screening in sexually-active PWH. Future studies should clarify whether syphilis-related lymphocyte depletion is non-subset-specific and occurs regardless of HIV-status.

**Table 1:** Characteristics of the syphilis episodes (N = 288)

Age, year	41 (33–48)
Male, n (%)	287 (99.7)
Nadir CD4 (cells/mm ^3^ )	197 (51–293)
Plasma viral suppression (< 50 copies/ml) at T1, n (%)	280 (97)
Duration between T1 & T2, days	168 (110–210)
Duration between T2 & syphilis treatment, days	16 (11–39)
Duration between syphilis treatment & T3, days	116 (84–175)
Syphilis re-infection (positive syphilis EIA at T1), n (%)	221 (77)
Treatment regimen, n (%) Penicillin Doxycycline Ceftriaxone Unknown	258 (90) 15 (5) 11 (4) 4 (1)

Median and IQR are reported unless specified. T1 = time-point before incident syphilis; T2 = time-point during syphilis; T3 = time-point after successful syphilis treatment. EIA = Enzyme immunoassay

**Table 2:** Plasma HIV-1 suppression, total white blood and various lymphocyte counts across syphilis

	T1	T2	T3	T1 vs. T2 p-value	T2 vs. T3 p-value
CD4 + T-cell count (cells/mm^3^ )	559 (405–699)	519 (390–687)	562 (430–725)	0.003	< 0.001
CD8 + -cell count (cells/mm^3^ )	822 (621–1047)	733 (547–954)	805 (621–1044)	< 0.001	< 0.001
CD4/CD8 ratio	0.67 (0.50–0.90)	0.71 (0.52–0.95)	0.70 (0.52–0.95)	< 0.001	0.542
Plasma viral suppression, n (%)	280 (97)	282 (98)	284 (99)	0.791	0.754
Total white blood count (10^3^ cells/µL)	5.9 (5.1–7.2)	6.2 (5.1–7.2)	6.1 (5–7.4)	0.557	0.970
Total lymphocyte count (10^3^cells/µL)	2.1 (1.7–2.6)	1.9 (1.6–2.4)	2.1 (1.8–2.6)	< 0.001	< 0.001

Median and IQR are reported unless specified. Parameters of continuous and categorical format were compared by Wilcoxon and McNemar test, respectively. T1 = time-point before incident syphilis; T2 = time-point during syphilis; T3 = time-point after successful syphilis treatment

## Supplementary Information

**Additional file 1: Table S1.** Univariable analysis betweenthe changes in lymphocyte levels from T1 to T2 and their potential determinants. 

## Data Availability

The datasets used and/or analyzed during the current study are available from the corresponding author on reasonable request.
