# Supplementary material for: Effects of syphilis infection among HIV-1-positive individuals on suppressive antiretroviral therapy
Source: AIDS Res Ther. 2022 Dec 31;19:69. doi: 10.1186/s12981-022-00493-w (PMC9805186; doi:10.1186/s12981-022-00493-w)
Supplement: Supplementary file 1 — Additional file 1: Table S1. Univariable analysis betweenthe changes in lymphocyte levels from T1 to T2 and their potential determinants. [file 12981_2022_493_MOESM1_ESM.docx]

**Supplementary Table.** Univariable analysis between the changes in lymphocyte levels from T1 to T2 and their potential determinants

| **Independent Factors** | **CD4+ T-cell** | | **CD8+ T-cell** | | **Total Lymphocytes** | |
| --- | --- | --- | --- | --- | --- | --- |
|  | **r** | **p-value** | **r** | **p-value** | **r** | **p-value** |
| Age | -0.079 | 0.181 | -0.056 | 0.345 | -0.024 | 0.683 |
| Nadir CD4+ T-cell count | -0.026 | 0.666 | 0.029 | 0.626 | 0.048 | 0.417 |
| Duration between Blood Samplings | 0.057 | 0.336 | 0.001 | 0.989 | 0.077 | 0.195 |
| VDRL titre (At diagnosis) | -0.118 | 0.045 | -0.167 | 0.004 | -0.136 | 0.021 |
| Pre-CD4/CD8 ratio | -0.074 | 0.208 | 0.189 | 0.001 | 0.118 | 0.046 |
|  |  |  |  |  |  |  |
| ^#^Syphilis reinfection  Yes  No | -24 (-102 to 56)  -25 (-118 to 76) | 0.992 | -64 (-212 to 41)  -91 (-334 to 53) | 0.186 | -0.1 (-0.4 to 0.1)  -0.3 (-0.7 to 0) | 0.037 |
| All tests are done using Spearman Correlation (two-tailed) unless specified  ^#^ Median (IQR); Non-parametric Mann-Withney U test | | | | | | |
